# Social Networking Sites (SNSs) in medical education: a student’s perspective

**DOI:** 10.1080/10872981.2018.1524689

**Published:** 2018-09-25

**Authors:** Rans Nadir, Khayam Bashir, Mustafa Abdulsalam Nasir, Hassan Ali Khan

**Affiliations:** School of Medicine, Imperial College London, London, UK; Faculty of Biology, Medicine and Health, University of Manchester, Manchester, UK

As fourth year medical students with an interest in medical education and avid users of Social Networking Sites (SNSs); we took great curiosity in the research published by Guraya SY et al. [1]. We appreciate how much more prevalent the use of social media has become in recent years, especially among young adults [2]. Naturally, this has had an impact on the way we go about our learning and on education in general.

In accordance with the study, we have also experienced that Facebook is the most widely used social media. However our experiences differ when it comes to its use in education as we have found it is widely used amongst us and our peers. Prior to beginning our first year, all students were invited by our respective universities to join a Facebook ‘group’ for our specific cohort. Since then, these groups have been used for sharing notes, giving helpful advice, as well as passing on information regarding upcoming lectures and seminars. We believe this is the strongest case for the use of SNSs in medical education as it provides a platform for any student to share their resources which are all compiled in one place. Perhaps if the universities where the study took place adopted a similar approach, SNSs would have been more widely used for educational purposes.

As discussed, past research indicates that a major downside of SNSs is the lack of actual social interaction [3]. This is especially relevant in the case of medicine due to its practical aspect. One possible way to mitigate this issue is the use of Facebook’s ‘Group Video Call’ feature. This provides a setting in which students can discuss learning agendas as well as practice key skills such as history taking for objective structured clinical examinations (OSCEs). A method we have devised is a ‘Group Video Call’ in which each student takes upon a role as one of: a candidate, a patient and an examiner. Although a real life scenario is impossible to replicate, this system has proven to be extremely useful when meeting in person becomes impractical. Similar virtual environments are already being used in other domains such as in job interviews and conference calls to great effect [4]. Therefore, further study into its effectiveness in medical education would be of great interest.

Another barrier that is suggested is the distracting and addictive nature of social media. Although the study explored several different SNSs, it neglected to explore students’ use of messaging based services such as ‘WhatsApp’ which are not strictly classed as SNSs. These provide a platform for communicating by text, voice or video call, but do not come with the added distractions that sites such as Facebook and Twitter do. There is indeed some evidence for the use of text messages in group learning environments [5]. A strategy we have found effective is using the ‘group chat’ feature where multiple members can post and view text messages. The messages can be explanations to complex ideas or helpful revision aids such as mnemonics.

Given our own experiences in using various forms of social media for our education and the observed ubiquity of SNSs in our generation, we believe there is an important part for them to play in the future. We would be curious to see the results if the study was replicated in western institutions which, in our experience, already promote the use of SNSs in education. Moreover, we believe this study would be greatly furthered if interventions were implemented to promote the use of SNSs. The students could be provided with a framework to share resources such as a ready-made Facebook ‘group’ or made aware of specific features, notably the ‘Group Video Call’. Lastly, another avenue to go down could be comparing the effectiveness of messaging services such as ‘WhatsApp’ with SNSs.
